# Dual Cross-Linking of Catechol-Alginate Hydrogels: A Strategy for Enhanced Stability and Sustained Drug Delivery

**DOI:** 10.1021/acsomega.5c00077

**Published:** 2025-03-18

**Authors:** Zi-Ting Feng, Wei-Bor Tsai

**Affiliations:** Department of Chemical Engineering, National Taiwan University, Taipei 106319, Taiwan

## Abstract

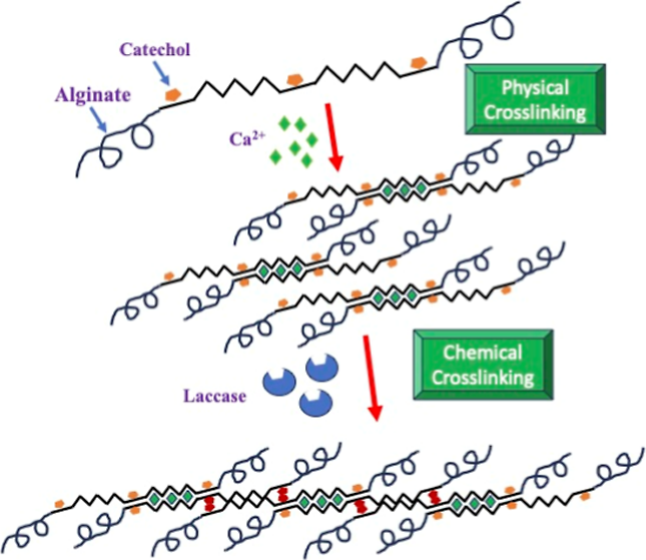

This study presents the development and characterization of a novel catechol-conjugated alginate (C-Alg) hydrogel system that combines Ca^2+^-mediated ionic cross-linking with laccase-catalyzed enzymatic cross-linking. While traditional Ca^2+^-mediated alginate hydrogels are known for their rapid gelation, they suffer from poor long-term stability in physiological conditions, limiting their effectiveness in medical applications. In the system developed here, the hydrogel initially formed upon mixing C-Alg with Ca^2+^, followed by gradual chemical catechol cross-linking through enzyme catalysis. The C-Alg hydrogels exhibited markedly improved mechanical strength and enhanced stability compared to those cross-linked with Ca^2+^ alone. This dual cross-linking approach also effectively slowed the release rates of model molecules with molecular weights ranging from several hundred to ten thousand. These advantages highlight the potential of C-Alg hydrogels as effective platforms for injectable drug delivery systems.

## 1 Introduction

Alginate, a linear polysaccharide derived from brown algae, consists of α-l-guluronic acid (G) and β-d-mannuronic acid (M) residues linked by 1,4-glycosidic bonds.^1^ Due to its excellent biocompatibility, low toxicity, and cost-effectiveness, alginate is widely used in biomedical engineering,^2^ similar to other polysaccharides. However, alginate stands out for its simple, rapid, and unique gelation mechanism. This process involves ionic cross-linking with divalent cations, such as calcium ions, which interact with the carboxylate anions in the G blocks of alginate, forming a structure known as the “egg-box” model.^3^ The ability of alginate to gel quickly under mild and nontoxic conditions makes it an attractive material for various biomedical applications, including wound dressings, tissue engineering, and drug delivery.^2,4−6^

Ca^2+^-mediated cross-linking of alginate, either alone or in combination with other biopolymers, enables the encapsulation of a wide range of drugs for diverse clinical therapies.^7−9^ However, one of the major challenges with Ca^2+^-mediated alginate hydrogels in drug delivery is achieving sustained drug release.^10^ Alginate/Ca^2+^ hydrogels typically dissolve within 1 to 2 h when immersed in physiological solutions, particularly those containing monovalent cations, leading to the rapid release of encapsulated drugs.^11^ This quick dissolution can result in undesirable side effects, reduced drug efficacy, and limited therapeutic duration. Additionally, the fragile and unstable network structure of these hydrogels restricts their use in advanced drug delivery systems. A potential solution to this issue is chemical cross-linking of alginate, either through its carboxylate groups^12,13^ or via aldehyde formation through oxidation of the polymer.^14−16^ However, relying solely on chemical cross-linking removes alginate’s unique benefit of Ca^2+^-mediated gelation, reducing alginate’s advantages over other natural hydrogels in drug encapsulation. Therefore, developing a novel alginate hydrogel that combines rapid gelation through Ca^2+^ cross-linking with covalent cross-linking for long-term stability would be an effective strategy to create a gelation system that achieves both immediate gelation and sustained drug release.

Alginate conjugated with catecholic groups could be chemically cross-linked via auto-oxidation.^17^ The chemical cross-linking of polysaccharides conjugated with phenolic or catecholic groups can be accelerated through the catalytic activity of various enzymes, such as horseradish peroxidase (HRP), tyrosinase, catalase, and laccase, via the oxidation of these functional groups.^18−20^ Alginate conjugated with catecholic groups can undergo oxidative cross-linking in the presence of oxidants^21^ and enzymatic cross-linking using HRP^22^ or laccase.^23^ However, HRP requires toxic H_2_O_2_ for the oxidative coupling of phenolic moieties,^18^ limiting its applicability in physiological conditions. In contrast, laccase, a multicopper oxidase, catalyzes the oxidation of various functional groups, including diphenols, methoxy-substituted monophenols, and aromatic amines, using nontoxic oxygen as the oxidant.^24^ It effectively facilitates the conjugation of phenolic molecules^25^ and has been reported to catalyze the oxidative polymerization of methoxyphenols.^26^ Laccase offers a biocompatible environment for cross-linking catechol-grafted polysaccharides to form hydrogels in biomedical applications.

The aim of this study was to develop an alginate-based hydrogel that is cross-linked simultaneously by both Ca^2+^-mediated and enzymatic mechanisms. To the best of our knowledge, no such alginate hydrogel has been developed before. Alginate was conjugated with dopamine to form catechol-conjugated alginate (C-Alg), which was capable of forming hydrogels via Ca^2+^, laccase, or a combination of both. The properties of C-Alg hydrogels were characterized to compare gelation through single and dual mechanisms. The release of model molecules with molecular weights ranging from several hundred to ten thousand was evaluated to simulate sustained drug release. Our results demonstrate the potential of these alginate hydrogels as effective drug carriers.

## 2 Materials and Methods

### 2.1 Materials

Alginic acid sodium salt isolated from brown algae (A0682), dopamine hydrochloride (H8502), *N*-3-(dimethylamino)propyl-*N*-ethyl carbodiimide hydrochloride (EDC, E7750), *N*-hydroxysuccinimide (NHS, 130672), calcium chloride (C4901), laccase from *Trametes versicolor* (38429), 2-morpholino-ethanesulfonique hydrate (MES, M2933), fluorescein sodium salt (FSS, F6377), and fluorescein isothiocyanate (FITC)–dextran (average molecular weight 4000 and 10,000 Da, 46944 & FD10S, respectively) were purchased from Sigma-Aldrich (St. Louis, USA). MES buffer (0.05 M, pH 6.0) was prepared by dissolving 9.762 g MES in 1 L of deionized water and the pH was adjusted to 6.

### 2.2 Synthesis and Characterization of Catechol Conjugated Alginate (C-Alg)

Catechol-conjugated alginate (C-Alg) was synthesized by grafting dopamine onto alginate using an EDC/NHS reaction. Alginate was dissolved in deionized water to a concentration of 2% w/v, and the pH of the solution was adjusted to 6.0. EDC and NHS were then added to the alginate solution, which was gently stirred for 45 min to activate the carboxyl groups on the alginate. Dopamine was subsequently added to the mixture at a molar ratio of 2.5:1 relative to the carboxyl groups of alginate. The reaction mixture was gently stirred for 12 h at room temperature under a constant nitrogen purge to prevent dopamine oxidation. After the reaction was complete, the C-Alg product was precipitated by adding a 10-fold excess of ethanol to the mixture. The precipitated C-Alg was then purified by redissolving it in deionized water and reprecipitating it in ethanol twice. Finally, the purified C-Alg was lyophilized to obtain the dried product.

Catechol conjugation was confirmed using ^1^H nuclear magnetic resonance (NMR, AVIII-500 MHz FT-NMR, Bruker, USA) and Fourier-transform infrared spectroscopy (FTIR, PerkinElmer Spectrum 100, in the 400–4000 cm^–1^ region). The degree of substitution (DS) of dopamine was determined by measuring the absorbance at 280 nm, which is characteristic of catecholic groups. The catechol content was calculated from a standard curve generated with varying concentrations of dopamine (see S1 in Supporting Information). The degree of substitution was then calculated as follows
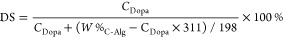
where *C*_Dopa_ is the catechol concentration in mmol/mL, *W* %_C-Alg_ is the mass concentration of the C-Alg sample in mg/mL, and 311 and 198 were the molecular weight of the substituted side chain units and the alginate units, respectively.

### 2.3 Formation and Evaluation of Gelation in Catechol-Conjugated Alginate Hydrogels

C-Alg hydrogels were formed through physical cross-linking with Ca^2+^, laccase-mediated cross-linking, or a combination of both, designated as C-Alg/Ca, C-Alg/L, and C-Alg/L/Ca, respectively. For hydrogel preparation, C-Alg was dissolved in MES buffer at a concentration of 2% w/v. This solution was then mixed with Ca^2+^, laccase (1 U/mL), or both, and poured into molds for gelation. The gelation time was defined as the duration required for the mixture to lose its fluidity when the vial was tilted to a 45-degree angle.

To evaluate the degree of gelation, 100 μL of the C-Alg/L or C-Alg/L/Ca solution, containing 2% (w/v) C-Alg and 1 U/mL laccase, with or without 7 mM Ca^2+^, was placed in a 7 mm diameter mold and left to gel for 10, 30, 50, or 70 min. The formed hydrogels were then immersed in phosphate-buffered saline (PBS) at pH 7.4 for 1 min to remove any unbound components. After immersion, the hydrogels were centrifuged to remove the supernatants. The remaining dry weight of each hydrogel was then measured to assess the extent of gelation and stability.

The percentage of remaining weight (*R*_w_) was calculated using the following equation: , where *W*_i_ and *W*_r_ were the initial and the remained weights of the hydrogel before and after immersion in PBS, respectively.

### 2.4 Assessment of Rheological and Compressive Properties of Hydrogels

The rheological and compressive properties of the hydrogels were assessed using a rheometer with a parallel plate geometry (20 mm diameter) (HR-2, TA Instruments, USA). During the oscillation time sweep measurements, the storage modulus (*G*′) and loss modulus (*G*″) were recorded at 1% strain and 1 Hz frequency over various gelation times, with each time point measured for 48 s. For the oscillation amplitude sweep measurements, the storage and loss moduli of fully gelled hydrogels were measured at a fixed frequency of 1 Hz. Axial compression tests were conducted on the fully gelled hydrogels, which were compressed at a rate of 36 mm/min until fracture. The Young’s modulus and bulk modulus were then calculated from the resulting compression curves.

### 2.5 Evaluation of Hydrogel Adhesiveness Using Lap Shear Stress Test on Porcine Skin

The adhesiveness of the hydrogels was evaluated using a lap shear stress test on porcine skin. The porcine skin was first washed and soaked in water to remove excess fat, then cut into rectangular strips (1.0 cm × 1.5 cm) and adhered to glass slides. A 50 μL sample of the hydrogel solution (Alg/Ca, C-Alg/Ca, C-Alg/L, or C-Alg/L/Ca) was applied between two pieces of porcine skin with a 1.0 cm × 1.0 cm overlap. The upper end of the adhered samples was pulled upward at a rate of 84 mm/min using a programmable force test stand (FGS-100PV, NIDEC-SHIMPO, Japan) until the two skin pieces separated. The force required to separate the porcine skin strips was recorded using a digital force gauge (FGP-0.5, NIDEC-SHIMPO, Japan) with a capacity of 5 N. Each test group was performed in quintuplicate.

### 2.6 Determination and Mathematic Fitting of the Release of Model Molecules from the Hydrogels

The release behaviors of model molecules from Alg/Ca, C-Alg/Ca, and C-Alg/L/Ca hydrogels were evaluated. A total of 5 μg of fluorescein sodium salt (FSS) or FITC-dextran, with molecular weights of 4 kDa and 10 kDa (abbreviated as FD4 and FD10, respectively), was mixed with 100 μL of hydrogel and loaded into a circular mold with a 7 mm diameter to form a thin hydrogel film. The formed hydrogel film was then immersed in 3 mL of PBS and gently shaken at room temperature. At specified time points, 2 mL of the PBS solution was collected for fluorescence measurement, and 2 mL of fresh PBS was added to replenish the solution. The fluorescence intensities of the collected samples were measured using a fluorescence microplate reader, with excitation and emission wavelengths set at 490 and 520 nm, respectively. The amounts of released model molecules were determined from standard curves generated using various concentrations of FSS or FITC-dextran.

The experimental data for the release of FSS and FITC-dextran were fitted to a mathematical model of drug release for thin film geometry^27^ using MATLAB software with the lsqcurvefit function.
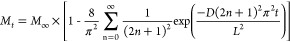
where *M*_*t*_ and *M*_∞_ are cumulative amounts of FSS or FTIC-dextran released at time *t* and infinity, respectively. *n* is a dummy variable; *D* is the diffusion coefficient of a molecule; *L* is the thickness of the film.

### 2.7 Statistical Analysis

Statistical analyses of the data were performed by Prism 9 (GraphPad, USA) using the one-way or two-way ANOVA followed by Tukey’s multiple comparison test. *, **, *** and **** indicate *p* value < 0.05, 0.01, 0.001 and 0.0001, respectively. All the values are presented as mean ± standard deviation.

## 3 Results and Discussion

### 3.1 Characterization and Quantification of Dopamine Conjugation of C-Alg

The conjugation of dopamine to alginate was confirmed using ^1^H NMR and FTIR spectroscopy. In the ^1^H NMR spectrum of alginate (see S2 in the Supporting Information), peaks between 3.6 and 4.2 ppm were attributed to the protons in the alginate backbone, while peaks around 4.5 and 5.1 ppm corresponded to the protons in the guluronate units. After dopamine conjugation, new peaks appeared in the C-Alg spectrum between 6.6 and 6.9 ppm, corresponding to the aromatic protons of the catechol moiety. Additionally, the peak at 7.0 ppm was associated with the protons of the amide bond, and the peaks at 2.8 and 3.2 ppm indicated the protons on the carbon adjacent to the amide bond. These NMR findings demonstrate the successful conjugation of dopamine to alginate.

In the FTIR spectrum of C-Alg (see S3 in the Supporting Information), the peaks at 1741 and 1528 cm^–1^ were attributed to N–H bending vibrations, while the peak at 1247 cm^–1^ corresponded to the phenolic structure of catechol. Together, the ^1^H NMR and FTIR spectra confirmed the successful conjugation of dopamine to alginate. The degree of dopamine conjugation was further quantified by measuring the absorbance at 280 nm. Using a standard curve, the degree of substitution was calculated to be 45%.

### 3.2 Influence of Ca^2+^ and Laccase on the Gelation and Stability of C-Alg Hydrogels

**Figure 1:**
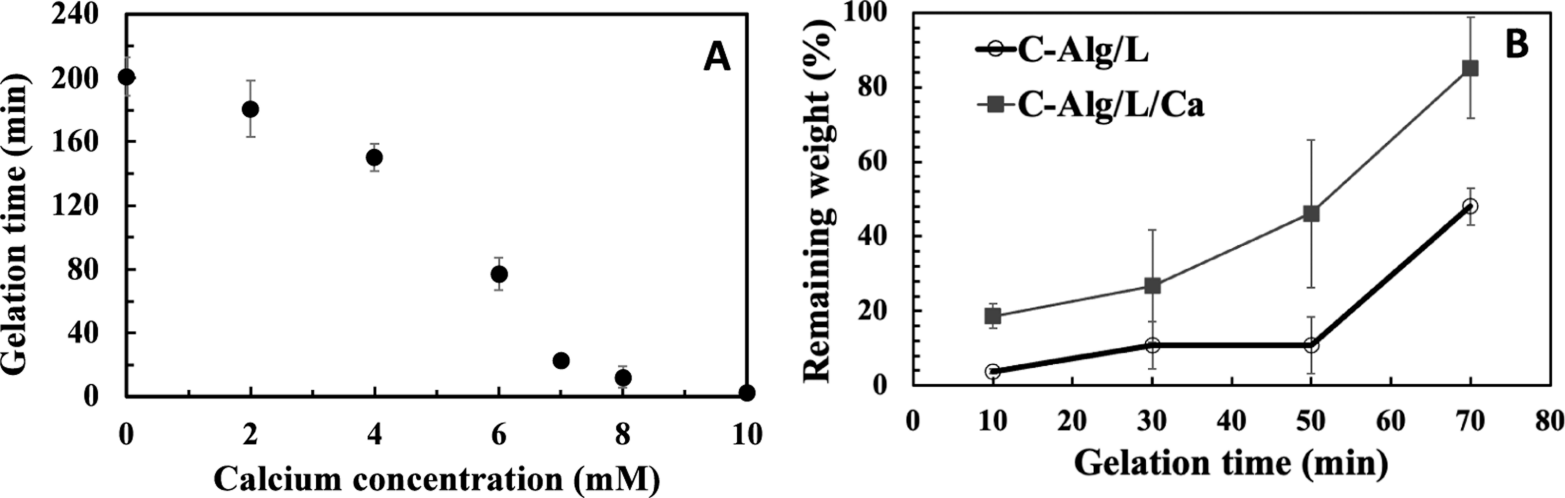
(A) Gelation time of 2% C-Alg solutions containing 1 U/mL laccase with varying concentrations of Ca^2+^. *n* = 3. (B) Stability assessment of C-Alg hydrogels cross-linked by 1 U/mL laccase with or without 7 mM Ca^2+^ after different gelation times in PBS. *n* = 3. Error bars represent the standard deviation.

The gelation of 2% C-Alg in the presence of Ca^2+^ and laccase was first investigated using a vial tilting assay, a practical, empirical method commonly used to assess the point at which a material transitions from a liquid to a semisolid state. The gelation time for C-Alg/L without Ca^2+^ was approximately 200 min, but this time decreased significantly with increasing Ca^2+^ concentrations, reaching around 20 min with 7 mM Ca^2+^ (Figure 1A). These results indicate that Ca^2+^ accelerates the gelation process through rapid ionic cross-linking. At Ca^2+^ concentrations higher than 7 mM, the gelation time was further reduced, reaching just 2 min at 10 mM. However, at such high Ca^2+^ concentrations, the C-Alg solution became too viscous to mix properly, resulting in a heterogeneous mixture with white fibrillar structures. Therefore, 7 mM Ca^2+^ was selected for subsequent experiments.

**Scheme 1:**
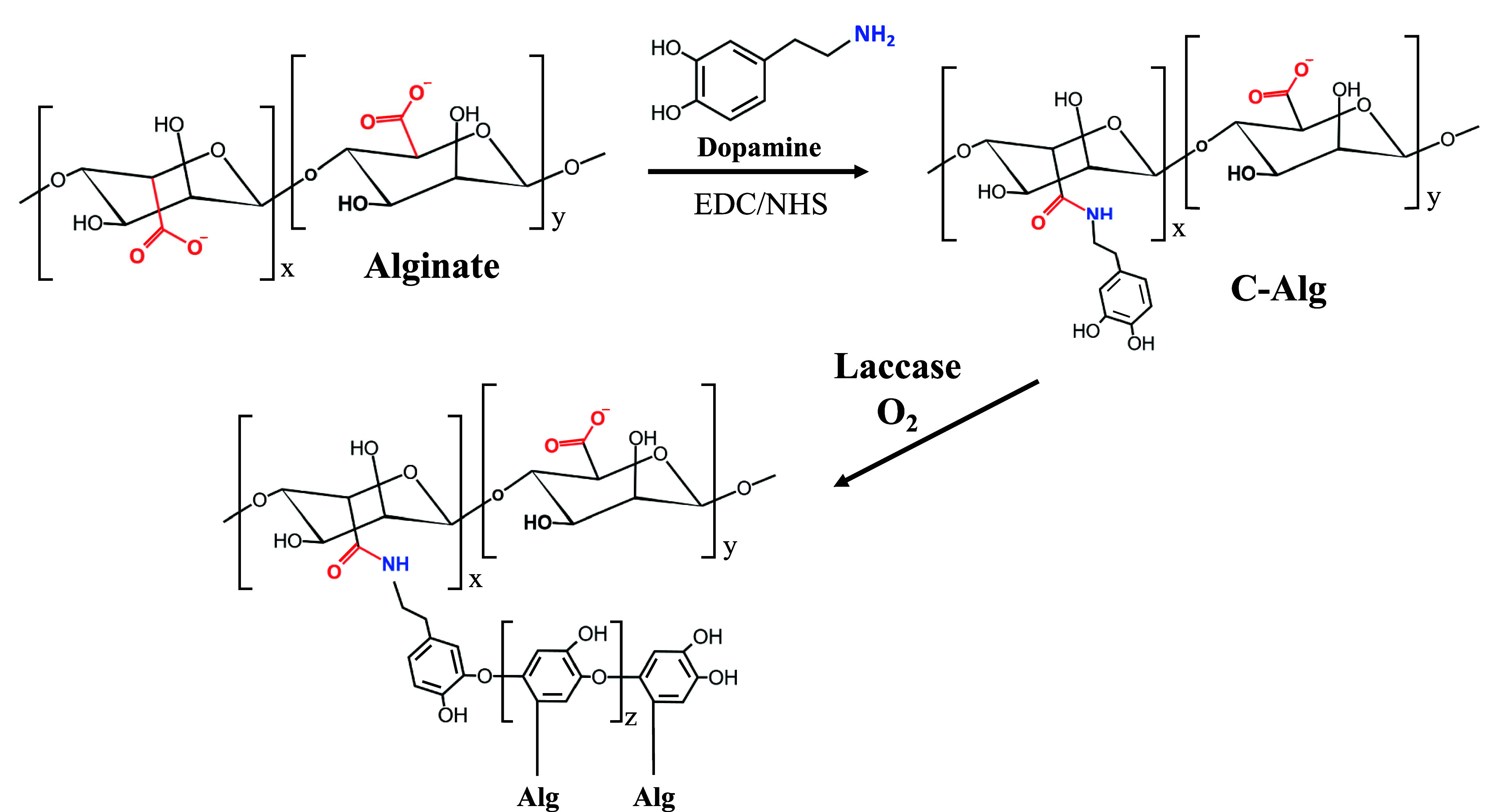
Proposed Mechanism of the Formation of C-Alg Hydrogel

Simply stopping the flow does not confirm complete gelation, so we further investigated the degree of gelation for C-Alg/L and C-Alg/L/Ca at different time points. The formed hydrogels were immersed in PBS to remove unbound C-Alg, and the remaining masses were weighed to estimate the degree of gelation. In the absence of laccase, C-Alg/Ca did not form stable hydrogel within the incubation time and rapidly dissolved in PBS. Conversely, the presence of laccase significantly enhanced the stability of C-Alg hydrogels, with stability increasing as gelation time progressed (Figure 1B). Without Ca^2+^, the C-Alg/L hydrogel failed to form a cohesive structure in PBS, resulting in considerable mass loss. In contrast, the C-Alg/L/Ca hydrogel maintained its bulk structure, retaining 85% of its mass after 70 min of gelation, compared to only 48% for C-Alg/L. These results indicate that Ca^2+^ plays a crucial role in supporting and enhancing the stability of laccase-catalyzed C-Alg hydrogels.

### 3.3 Assessment of Rheological and Compressive Properties of Alg and C-Alg Hydrogels

Although the tilt test is a convenient and qualitative method for estimating gelation time, it does not accurately define the gelation process from a physicochemical perspective. Thus, we further determined the gelation processes of the Alg and C-Alg hydrogels using rheological oscillation time sweep tests. The gel point, which marks the transition from liquid-like to solid-like behavior, is identified at the crossover of the storage modulus (*G*′) and the loss modulus (*G*″). The hydrogels containing Ca^2+^, Alg/Ca, C-Alg/Ca, and C-Alg/L/Ca, formed gels immediately upon mixing, as indicated by *G*′ being higher than *G*″ from the start of the tests (see S4 of Supporting Information). In contrast, the gel point for C-Alg/L appeared approximately 2 h after mixing.

**Figure 2:**
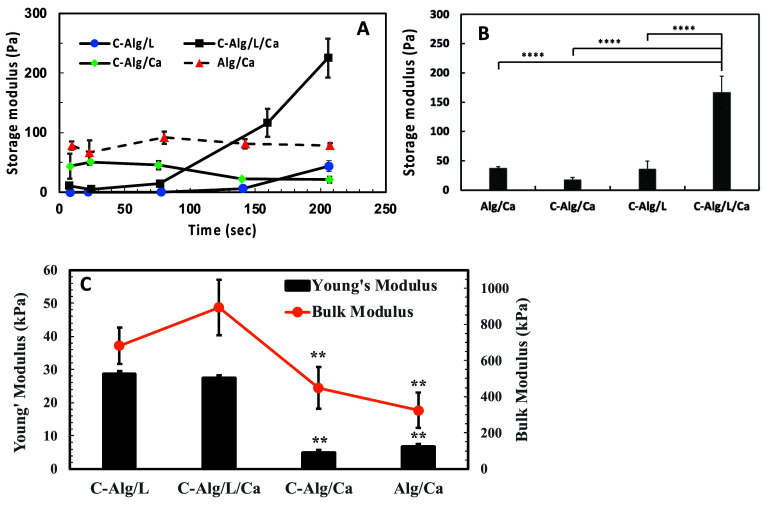
Rheological and compressive properties of 2% Alg and C-Alg hydrogels with or without addition of Ca^2+^ and laccase. (A) Oscillation time sweep measurement at different gelation times. (B) Oscillation amplitude sweep measurement for completely gelled hydrogels. *n* = 3. **** represents *p* < 0.0001 vs the other groups. (C) Young’s and bulk modulus of different types of hydrogels. *n* = 3. ** represent *p* < 0.01 vs C-Alg/L/Ca.

The storage modulus (*G*′) reflects the mechanical strength of the hydrogels. At the initial time point (∼8 min after mixing), the *G*′ of Alg/Ca was 78.5 Pa, while that of C-Alg/Ca was 43.8 Pa (Figure 2A). The reduction in *G*′ for C-Alg/Ca is likely due to the conjugation of guluronic carboxylates with catechol, which reduces its interaction with Ca^2+^. The addition of laccase further decreased the *G*′ to 11.2 Pa for C-Alg/L/Ca, suggesting that laccase interferes with the interaction between C-Alg and Ca^2+^. Without Ca^2+^, the *G*′ of C-Alg/L was only 0.02 Pa.

As the gelation process continued, the *G*′ of Alg/Ca remained relatively constant, while that of C-Alg/Ca gradually decreased from 43.8 to 22 Pa over 2 h (Figure 2A). Conversely, the mechanical strength of C-Alg hydrogels containing laccase increased over time. The *G*′ of C-Alg/L rose steadily from 0.02 to 43.9 Pa after 200 min, eventually surpassing that of C-Alg/Ca. These findings indicate that chemical cross-linking via laccase produces stronger C-Alg hydrogels than those formed by Ca^2+^ cross-linking alone. Combining both Ca^2+^ and laccase cross-linking resulted in the strongest hydrogels. The *G*′ of C-Alg/L/Ca reached 225 Pa after 200 min of gelation, significantly higher than that of Alg/Ca. These results suggest that Ca^2+^ facilitates the enzymatic cross-linking process, possibly by bringing catechol groups closer together, thereby enhancing laccase activity and resulting in a stronger hydrogel network.

The storage modulus (*G*′) of fully gelled hydrogels was measured using oscillation amplitude sweep, as shown in Figure 2B. The C-Alg/L/Ca hydrogel exhibited a significantly higher storage modulus of 166.4 Pa, which is 4.6 times greater than that of the C-Alg/L hydrogel, which measured at 35.7 Pa. These results support the previous conclusion that the presence of Ca^2+^ enhances the chemical cross-linking of C-Alg, resulting in a stronger hydrogel.

The compressive Young’s moduli of C-Alg/L and C-Alg/L/Ca hydrogels were both approximately 28 kPa, significantly higher than those of the hydrogels without chemical cross-linking (C-Alg/Ca and Alg/Ca), as shown in Figure 2C. Similarly, the compressive bulk moduli of C-Alg/L and C-Alg/L/Ca were beyond 680 kPa, compared to less than 500 kPa for C-Alg/Ca and Alg/Ca hydrogels. These increases in both Young’s modulus and bulk modulus for the C-Alg/L and C-Alg/L/Ca hydrogels indicate enhanced elasticity and stiffness, demonstrating that chemical cross-linking substantially improves the mechanical properties of the hydrogels.

### 3.4 Assessment of Adhesiveness of Hydrogels to Porcine Skin

**Figure 3:**
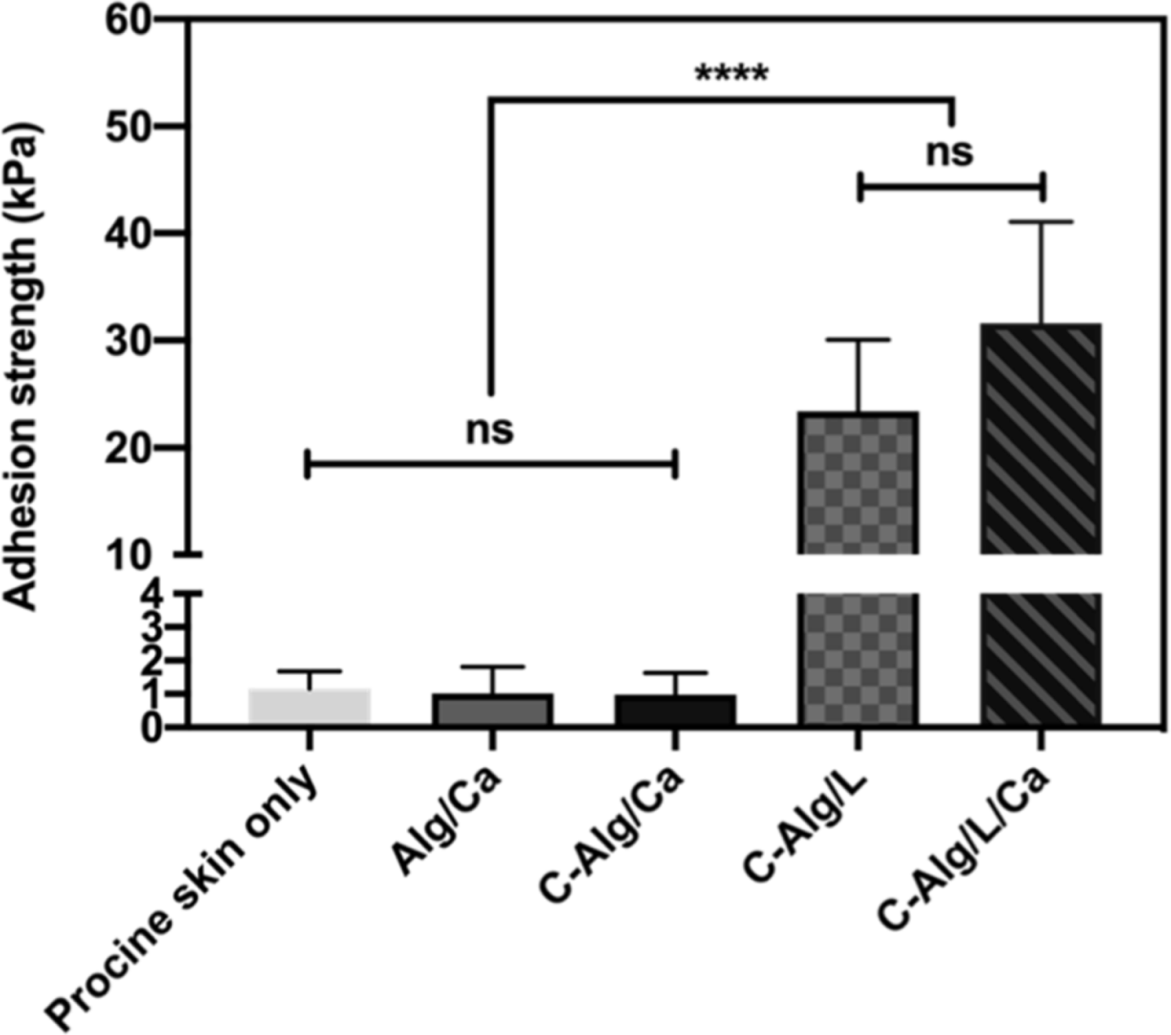
Adhesive strength between the porcine skins generated by different types of 2% alginate hydrogels. *n* = 5. **** represents *p* < 0.0001 compared with the groups. No significant difference is represented by ns.

The adhesive properties of hydrogels are crucial for their application to tissues. Catecholic groups have been shown to bond effectively to tissues through a mussel-inspired mechanism,^28^ involving various interactions such as hydrogen bonding, metal chelation, and π–π and/or cation–π interactions with tissue surfaces.^29^ Although the primary function of catechol in C-Alg in this study was to generate chemical cross-linking for hydrogel formation, it also imparted adhesive properties to tissues. To evaluate this, we tested the adhesiveness of C-Alg hydrogels on porcine skin. The results showed that Alg/Ca and C-Alg/Ca hydrogels exhibited no significant adhesiveness, similar to the porcine skin alone (Figure 3). In contrast, both C-Alg/L and C-Alg/L/Ca hydrogels demonstrated more than a 10-fold increase in adhesive strength. These findings suggest that while noncovalent interactions provide minimal adhesion, the laccase-catalyzed reaction significantly enhances the adhesive force between C-Alg and porcine skin. Furthermore, catechol cross-linking has been reported to increase the cohesion of catechol-containing adhesives,^30^ further improving their adhesive properties. One thing to be noted that we observed that residual hydrogels were present on both pieces of porcine skin after separation. This suggests that the hydrogel fractured within its bulk structure rather than detaching from the skin surface, indicating that the adhesive strength between the hydrogel and the porcine skin was stronger than the cohesive strength within the hydrogel itself.

### 3.5 Determination and Mathematic Fitting of the Release of Model Molecules from the C-Alg Hydrogels

Hydrogels are widely utilized as vehicles for drug delivery, and the release profile of a drug from a hydrogel is critical for achieving effective therapeutic outcomes. In this study, we investigated the release profiles of several model molecules with varying molecular weights: FSS (376 Da), 4 kDa FITC-dextran (FD4), and 10 kDa FITC-dextran (FD10), from Alg/Ca, C-Alg/Ca, and C-Alg/L/Ca hydrogels. These molecules, with different molecular weights, represent a range of small-molecule drugs to larger peptide or protein drugs.

**Figure 4:**
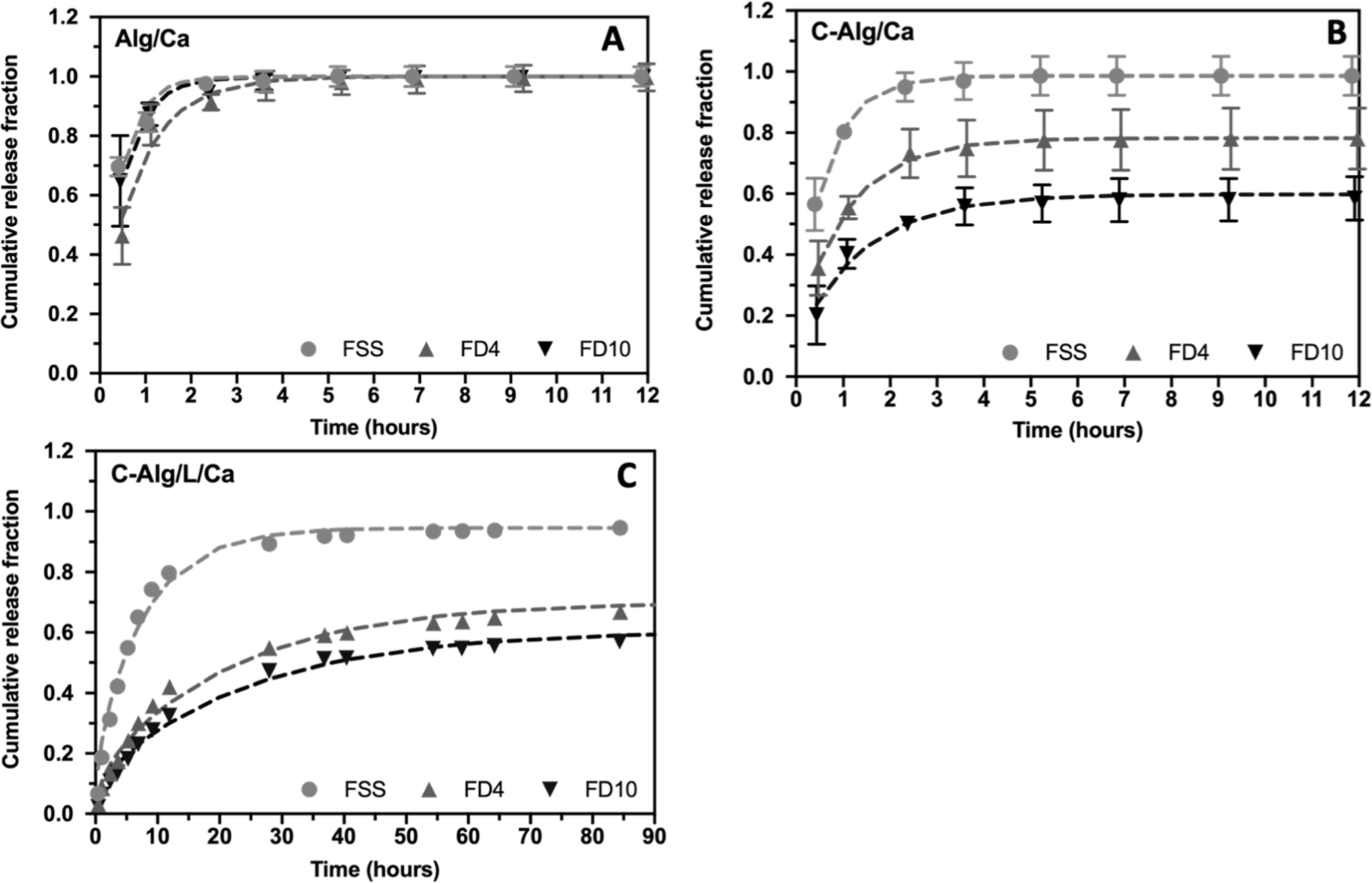
Release profiles of FSS and FITC-dextran (FD4 & FD10) from three types of hydrogels (2% w/v): (A) Alg/Ca, (B) C-Alg/Ca and (C) C-Alg/L/Ca hydrogel. *n* = 3. The dashed lines represent the fitting curves using the thin film model.

The release of all molecules from the Alg/Ca hydrogel was rapid, as illustrated in Figure 4A. Within the first hour, 90% of FSS and FD4 were released, while FD10 exhibited a slightly slower release, with 90% being released within 2.5 h. These results indicate the rapid disintegration of the Alg/Ca hydrogel, leading to a fast drug release, which is consistent with previous findings on the swift degradation and drug release characteristics of Alg/Ca hydrogels.^11^

In comparison, the release of FSS from the C-Alg/Ca hydrogel was slower, with 90% released within the first 2 h (Figure 4B). The release rates of FD4 and FD10 were even more gradual, reaching a plateau at approximately 70% and 50% after 2 h, respectively. As shown earlier in Figure 2, the mechanical properties of the C-Alg/Ca hydrogel were the weakest among all the hydrogels tested, indicating a low degree of cross-linking. This would typically suggest a rapid drug release. However, the slower release from the C-Alg/Ca hydrogel compared to the Alg/Ca hydrogel may be attributed to the self-polymerization of catechol groups, even in the absence of laccase, or the interaction of catechol groups with the model molecules.

In contrast to both Alg/Ca and C-Alg/Ca hydrogels, the C-Alg/L/Ca hydrogel maintained its integrity in PBS throughout the release experiment, demonstrating enhanced structural stability due to enzymatic cross-linking. The release of the model molecules was significantly slowed (Figure 4C). It took 12 h for 80% of FSS to be released, while the release of FD4 and FD10 was extended beyond 48 h, eventually reaching a steady state at 60% and 45% release, respectively. A substantial portion of FD4 and FD10 remained entrapped within the hydrogel, likely due to the confinement of these larger molecules within the highly cross-linked network.

Further analysis of the release data using a thin film model allowed us to calculate the diffusivity of the molecules in different hydrogels, as shown in Table 1. The diffusivity values for FSS, FD4, and FD10 in the 2% Alg/Ca hydrogel were 396.06, 208.47, and 338.49 μm^2^/s, respectively. In the C-Alg/Ca hydrogel, the diffusivity of these molecules was lower than in the Alg/Ca hydrogel and decreased with increasing molecular weight, with values of 286.65, 173.75, and 131.99 μm^2^/s. The diffusivity values in the C-Alg/L/Ca hydrogel were more than an order of magnitude lower than those in both the Alg/Ca and C-Alg/Ca hydrogels. Moreover, the diffusivity in the C-Alg/L/Ca hydrogel also decreased with increasing molecular weight, with values of 23.49, 11.15, and 9.91 μm^2^/s for FSS, FD4, and FD10, respectively. These results indicate that the chemical cross-linking in the C-Alg/L/Ca hydrogel creates a dense matrix that significantly hinders molecular diffusion, resulting in a controlled and sustained release.

**Table 1:** Diffusivity (D) of FSS and FITC-Dextran Fitted From Figure 4 Using a Thin Film Model

		FSS	FD4	FD10
Alg/Ca	D (μm^2^/s)	396.06	208.47	338.49
	*R*^2^	0.933	0.954	0.977
C-Alg/Ca	D (μm^2^/s)	286.65	173.75	131.99
	*R*^2^	0.995	0.996	0.975
C-Alg/L/Ca	D (μm^2^/s)	23.49	11.15	9.91
	*R*^2^	0.985	0.994	0.995

**Figure 5:**
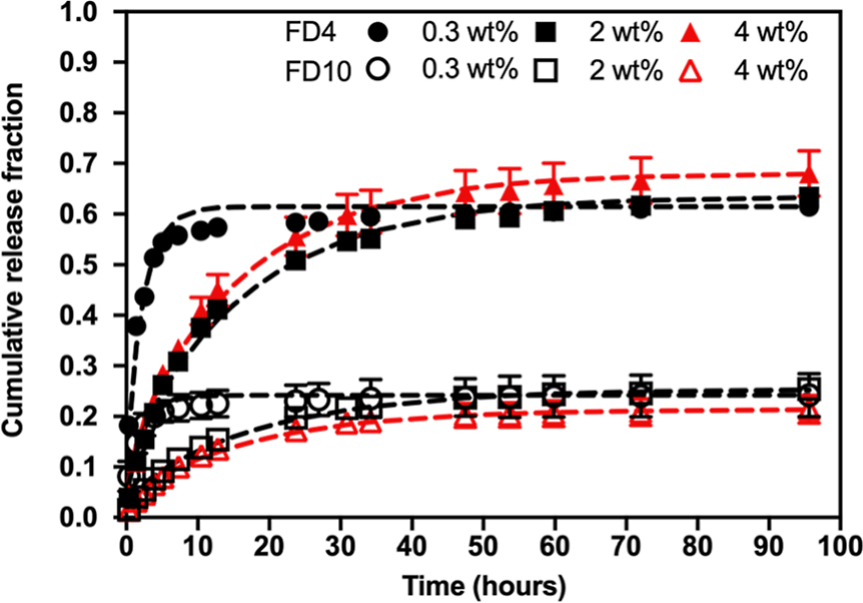
Release profiles of FITC-dextran (FD4 and FD10) from 0.3, 2, and 4 wt % C-Alg/L/Ca hydrogels. *n* = 3. The dashed lines represent the fitting curves using the thin film model.

We next evaluated the release of FITC-dextran (FD4 and FD10) from C-Alg/L/Ca hydrogels with varying C-Alg concentrations (0.3%, 2%, and 4%) (Figure 5). For FD4, the release profiles from the hydrogels of all three concentrations reached a plateau of approximately 60%, but at different times: 7, 40, and 65 h for 0.3%, 2% and 4% hydrogel with the diffusivity values were 81.10, 11.15, and 11.61 μm^2^/s, respectively (Table 2). For FD10, the release from 0.3% and 2% hydrogels reached a plateau of 23% after 10 and 50 h, respectively, while the release from 4% hydrogel plateaued of 17% after 50 h. The diffusivity values were 83.07, 9.91, and 10.72 μm^2^/s for FSS, FD4 and FD10, respectively (Table 2). These results indicate that even a low concentration (0.3%) of C-Alg/L/Ca hydrogel significantly reduced the release rate and amount of FD4 and FD10 compared to the 2% Alg/Ca hydrogel. The diffusivities of FD4 and FD10 in all three hydrogel concentrations were similar, with different plateaus.

**Table 2:** Diffusivity (*D*) Values of FD4 and FD10 Released From C-Alg/L/Ca Hydrogel as Shown in Figure 5

molecules		FD4	FD10
0.3 wt %	D (μm^2^/s)	81.10	83.07
	*R*^2^	0.953	0.942
2 wt %	D (μm^2^/s)	11.15	9.91
	*R*^2^	0.994	0.995
4 wt %	D (μm^2^/s)	11.61	10.72
	*R*^2^	0.994	0.993

Our results reveal an intriguing trend: the diffusivity of FD4 and FD10 is primarily influenced by the concentrations of C-Alg, while their retention is predominantly governed by their molecular sizes. It is generally accepted that the diffusivity and retention of drugs in hydrogels are controlled by both the network density (pore size) of the hydrogel and the molecular size of the drug. Specifically, increasing the concentration of C-Alg is expected to reduce the pore size by forming a denser polymer network, which theoretically should decrease diffusivity and enhance the retention of FD4 and FD10. However, our data indicate that retention is not influenced by C-Alg concentrations, suggesting that pore size differences are not the main factor determining retention.

This inconsistency suggests that the retention mechanism may involve interactions between catechol groups and the dextran backbone, potentially influenced by laccase activity. Catechol hydroxyl groups are known to form hydrogen bonds with the hydroxyl groups present on polysaccharide chains,^31^ enhancing adhesion and cohesion. Furthermore, laccase catalyzes the oxidation of catechol groups to quinones using molecular oxygen as the electron acceptor. These quinones are highly reactive and can undergo nucleophilic addition reactions with functional groups on polysaccharides, such as hydroxyl or amine groups.^32^

This mechanism suggests that FITC-dextran may gradually form covalent bonds with C-Alg, thereby enhancing its retention within the hydrogel. Additionally, these interactions are likely to become more pronounced with increasing molecular weight of FITC-dextran, potentially explaining the observed differences in retention. This insight highlights a limitation for drugs whose release profiles may be affected by laccase-mediated catechol reactions, providing valuable considerations for future applications.

Building on the promising outcomes observed with C-Alg hydrogels, it is valuable to place these findings within the broader context of catechol-functionalized hydrogels inspired by mussel adhesive proteins. Hydrogels can be formed from catechol-functionalized polymers through several mechanisms, including catechol oxidation, catechol–metal coordination, catechol–boronate complex formation, and catechol–thiol reactions.^33^ For instance, catechol-functionalized chitosan hydrogels have been developed for drug delivery in buccal and colonic applications.^34,35^ Furthermore, catechol-based hydrogels have been cross-linked with other agents, such as genipin^35^ or oxidized polysaccharides,^34^ to enhance mucoadhesive and mechanical properties. In this study, we developed a novel adhesive hydrogel by combining Ca^2+^-mediated cross-linking of alginate with laccase-catalyzed catechol cross-linking. The Ca^2+^/alginate interaction provides instant gelation, while the slow enzymatic cross-linking ensures long-term stability, making the hydrogel suitable for injection as a local drug delivery system with adhesiveness. The C-Alg hydrogel forms instantly upon mixing with a calcium solution, and its cross-linking strength gradually increases over time due to the laccase-catalyzed catechol reaction. To the best of our knowledge, this is the first instance of creating an alginate hydrogel combining both ionic and enzymatic cross-linking, thereby expanding the potential applications of alginate hydrogels.

This alginate gelation system also enhances the control of drug release. The release profiles of FSS, FD4, and FD10 from the Alg/Ca^2+^ hydrogel were similar, with complete release occurring within 4 h (Figure 4A). In contrast, the release of FSS from the Alg/L/Ca^2+^ hydrogel was much slower than that of FD4 and FD10, taking 40 h to reach a plateau (Figure 4C). The release of FD4 and FD10 could be modulated by varying the concentration of the hydrogels (Figure 5). However, a significant portion of FD4 and FD10 remained unreleased in the Alg/L/Ca^2+^ hydrogel, likely encapsulated within the cross-linked hydrogel. To release the remaining molecules, the hydrogel must be degraded.

We propose two approaches to facilitate the degradation of the C-Alg/L/Ca^2+^ hydrogel. One approach is periodate oxidation of alginate, which cleaves the carbon–carbon bond of the cis-diol group, thereby degrading its backbone.^36^ Additionally, dopamine can be conjugated to oxidized alginate, containing aldehyde groups, to form hydrazone bonds that are more susceptible to hydrolysis^37^ compared to the amide linkages used in this study. We plan to further investigate the controllable degradation of C-Alg hydrogels in future research.

## 4 Conclusion

This study has demonstrated the successful development of a catechol-conjugated alginate hydrogel system that integrates the rapid gelation of Ca^2+^-mediated ionic cross-linking with the sustained, gradual stabilization provided by laccase-catalyzed enzymatic cross-linking. The innovative combination of these two cross-linking mechanisms not only addresses the long-standing challenge of poor long-term stability in alginate hydrogels but also introduces a tunable platform for controlled and prolonged drug release. The enhanced mechanical properties and stability of these hydrogels suggest their strong potential for use in a variety of medical applications, particularly as injectable drug delivery systems that require both immediate structural integrity and sustained therapeutic action. Moving forward, we plan to explore the degradation kinetics of these hydrogels and to investigate their performance with a broader range of therapeutic agents, further establishing their versatility and efficacy in clinical settings.

## Supporting Information Available

A standard curve for quantification of dopamine conjugation; S2. The ^1^H NMR spectra of alginate and C-Alg; S3. The FTIR spectra of alginate and C-Alg; S4. The oscillation time sweep measurements of hydrogels (PDF)
