# Supplementary material for: Dual Cross-Linking of Catechol-Alginate Hydrogels: A Strategy for Enhanced Stability and Sustained Drug Delivery
Source: ACS Omega. 2025 Mar 18;10(12):12505–13. doi: 10.1021/acsomega.5c00077 (PMC11966316; doi:10.1021/acsomega.5c00077)

Supporting Information

Dual Crosslinking of Catechol-Alginate Hydrogels: A Strategy for Enhanced Stability and Sustained Drug Delivery

Zi-Ting Feng[1, 2], Wei-Bor Tsai[1,*]

S1. A standard curve for quantification of dopamine conjugation.

The degree of substitution (DS) of dopamine was determined by measuring the absorbance at 280 nm, which is characteristic of catecholic groups. The catechol content was calculated from a standard curve generated with varying concentrations of dopamine. A standard curve is shown below.

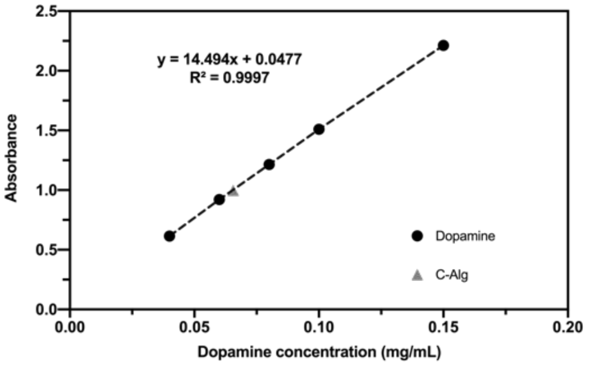

S2. The ^{1}H-NMR spectra of alginate and C-Alg

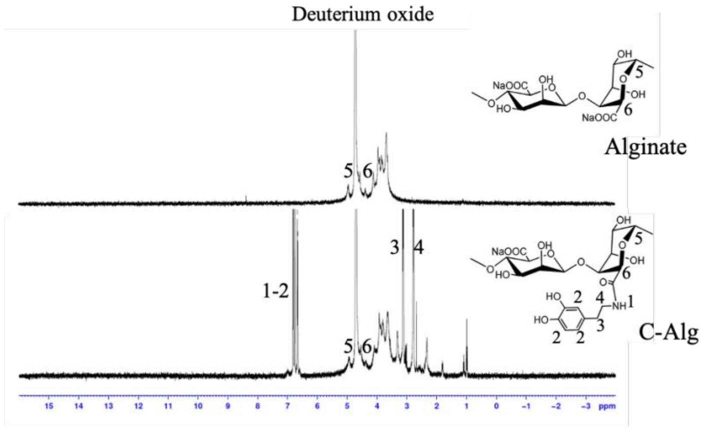

S3. The FTIR spectra of alginate and C-Alg

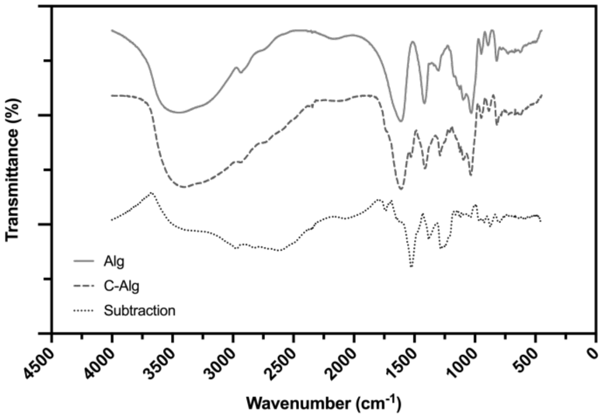

S4 The oscillation time sweep measurements of hydrogels

The rheological properties of the hydrogels were assessed using a rheometer with a parallel plate geometry (20 mm diameter) (HR-2, TA Instruments, USA). During the oscillation time sweep measurements, the storage modulus (G') and loss modulus (G") were recorded at 1% strain and 1 Hz frequency over various gelation times, with each time point measured for 48 seconds. (A) Alg/Ca; (B) C-Alg/L; (C) C-Alg/Ca; (D) C-Alg/L/Ca

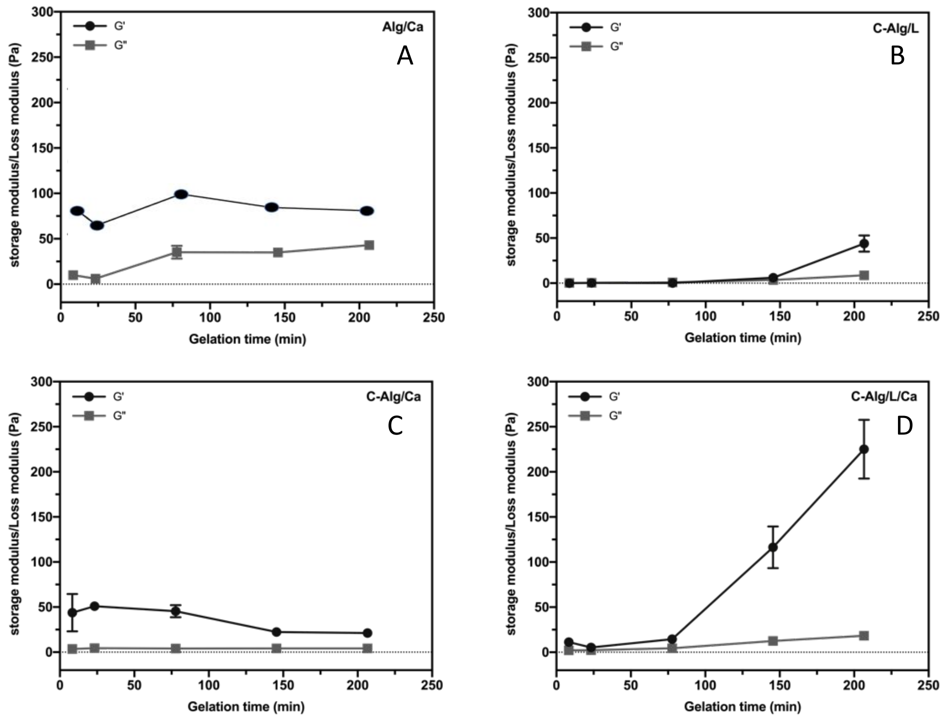

Supplement: Supplementary file 1 — ao5c00077_si_001.pdf [file ao5c00077_si_001.pdf]
